# Evidence that Memantine Reduces Chronic Tinnitus Caused by Acoustic Trauma in Rats

**DOI:** 10.3389/fneur.2012.00127

**Published:** 2012-08-15

**Authors:** Yiwen Zheng, Emily McNamara, Lucy Stiles, Cynthia L. Darlington, Paul F. Smith

**Affiliations:** ^1^Department of Pharmacology and Toxicology, Brain Health Research Centre, School of Medical Sciences, University of OtagoDunedin, New Zealand

**Keywords:** acoustic trauma, tinnitus, memantine, NMDA receptors, rats

## Abstract

Subjective tinnitus is a chronic neurological disorder in which phantom sounds are perceived. Increasing evidence suggests that tinnitus is caused by neuronal hyperactivity in auditory brain regions, either due to a decrease in synaptic inhibition or an increase in synaptic excitation. One drug investigated for the treatment of tinnitus has been the uncompetitive *N*-methyl-d-aspartate (NMDA) receptor antagonist, memantine, although the evidence relating to it has been unconvincing to date. We re-investigated the effects of memantine on the behavioral manifestations of tinnitus induced by acoustic trauma (a 16-kHz, 110-dB pure tone presented unilaterally for 1 h) in rats. We used a conditioned lick suppression model in which lick suppression was associated with the perception of high frequency sound resembling tinnitus and a suppression ratio (SR) was calculated by comparing the number of licks in the 15-s period preceding the stimulus presentation (A) and the 15-s period during the stimulus presentation (B), i.e., SR = B/(A + B). Acoustic trauma resulted in a significant increase in the auditory brainstem-evoked response (ABR) threshold in the affected ear (*P* ≤ 0.0001) and a decrease in the SR compared to sham controls in response to 32 kHz tones in five out of eight acoustic trauma-exposed animals. A 5-mg/kg dose of memantine significantly reduced the proportion of these animals which exhibited tinnitus-like behavior (2/5 compared to 5/5; *P* ≤ 0.006), suggesting that the drug reduced tinnitus. These results suggest that memantine may reduce tinnitus caused by acoustic trauma.

## Introduction

Subjective tinnitus has been estimated to occur in 25.3% of people in the USA, with 7.9% experiencing it frequently (Shargorodsky et al., 2010). Among other options, drugs are one of the main treatment avenues for severe tinnitus. However, despite the number of different drugs that are sometimes used for the condition, including benzodiazepines, anti-epileptic drugs, anti-spastic drugs, and even herbal medicines, there is surprisingly little convincing evidence to support their efficacy in consistently alleviating the condition (see Darlington and Smith, 2007; Elgoyhen and Langguth, 2011 for reviews).

Data from animal and human studies have suggested that tinnitus is associated with neuronal hyperactivity at different levels of the central auditory pathways, including the dorsal cochlear nucleus, the inferior colliculus, auditory cortex, and the striatum (see Moller, 2000; Eggermont and Roberts, 2004; Eggermont, 2005; Kaltenbach, 2006; Roberts et al., 2010 for reviews; see Dong et al., 2010; Rauschecker et al., 2010; Middleton et al., 2011; Mulders and Robertson, 2011; Vogler et al., 2011; Leaver et al., 2011 for recent examples). For this reason, drugs that increase synaptic inhibition, such as benzodiazepines and GABA_B_ receptor agonists, have been one avenue of investigation for potential new treatments for tinnitus (Middleton et al., 2011; Wang et al., 2011; Zheng et al., 2012; see Darlington and Smith, 2007; Elgoyhen and Langguth, 2011 for reviews). However, the mechanisms underlying the neuronal hyperactivity associated with tinnitus are not entirely understood, and drugs that block excitatory synaptic neurotransmission may also be effective. For example, there is some evidence that salicylate-induced tinnitus may involve an increase in glutamatergic neurotransmission at the *N*-methyl-d-aspartate (NMDA) subtype of glutamate receptor in the cochlea (Lin, 1997; Guitton et al., 2003) and that NMDA receptor antagonists can block this effect (Guitton et al., 2003). Moreover, long-term tinnitus induced by acoustic trauma was prevented by locally applying another NMDA receptor, polyamine site, antagonist, ifenprodil, into the cochlea within the first 4 days after the acoustic trauma (Guitton and Dudai, 2007).

Memantine is an uncompetitive antagonist at the NMDA receptor, which has been investigated as a potential treatment for tinnitus. Although it has a similar site of action in the NMDA receptor calcium channel to dizocilpine maleate (MK-801), its different channel kinetics confer upon it a more favorable adverse side effect profile (Olivares et al., 2012). However, limited studies of the effects of memantine on tinnitus have shown complex results. Lobarinas et al. (2006) examined its effects on salicylate- and quinine-induced tinnitus in rats and found that it failed to reduce the behavioral indices of tinnitus and the increase in cortical auditory-evoked potentials caused by salicylate (see also Lobarinas et al., 2008). In what appears to be the only published clinical trial, Figueiredo et al. (2008) studied the effects of memantine in 60 patients with tinnitus. This was a randomized, double-blind, placebo-controlled cross-over trial in which tinnitus severity was quantified using the Tinnitus Handicap Inventory (THI). However, 20 mg memantine per day for 90 days had no significant effect on the patients’ tinnitus compared to placebo. More recently, Suckfull et al. (2011) have reported some beneficial effect in tinnitus patients of neramexane, which is also an NMDA receptor antagonist. However, it also acts at the α_9_α_10_ nicotinic acetylcholine and 5-HT_3_ receptors and it is not clear which of its various actions might be responsible for its effects on tinnitus (Suckfull et al., 2011).

Given the small amount of evidence relating to memantine in the context of tinnitus and the fact that the drug is currently approved for the treatment of moderate to severe Alzheimer’s disease (Olivares et al., 2012) and is therefore available clinically, we considered it necessary to re-investigate the potential of memantine to treat tinnitus, using an acoustic trauma animal model in which tinnitus was indicated by a reduced suppression ratio (SR) in a conditioned lick suppression paradigm (Zheng et al., 2011a,b,c, 2012).

## Materials and Methods

### Subjects

Data were obtained from 16 male Wistar rats (300–350 g at the beginning of the study) divided into acoustic trauma (*n* = 8) and sham control (*n* = 8) groups. The animals were maintained on a 12:12 h light:dark cycle at 22°C and were water deprived throughout the tinnitus behavioral tests. All procedures were approved by the University of Otago Committee on Ethics in the Care and Use of Laboratory Animals.

### Drug administration

Memantine (Sigma) was dissolved in saline and animals received a daily 5 mg/kg s.c. injection 1 h before being tested with the conditioned lick suppression paradigm (see below), using a counterbalanced design. This dose of memantine was based on our own pilot experiments. Animals were first tested in the paradigm at 2 weeks following the acoustic trauma to confirm the presence of tinnitus and then tested following the vehicle (saline), 5 mg/kg memantine as well as during the washout of the drug. Each phase (pre-drug, vehicle, 5 mg/kg memantine, and washout) took 18 days of testing. Note that the counterbalanced design meant that noise trauma and sham animals received the drug and vehicle treatments in different orders and that the condition in which the noise trauma animals received the drug vehicle served as a control group for the condition in which the noise trauma animals received the drug.

### Noise trauma to induce chronic tinnitus in rats

Unilateral acoustic trauma was delivered using a procedure described previously (Bauer and Brozoski, 2001; Brozoski et al., 2007; Zheng et al., 2011a,b,c, 2012). Briefly, the animals were anesthetized with ketamine HCl (75 mg/kg, s.c.) and medetomidine hydrochloride (0.3 mg/kg, s.c.) and were placed inside a sound attenuation chamber for a 1 h exposure to a 16 kHz, 110 dB sound pressure level (SPL) pure tone delivered to one of the ears as described in detail previously (Zheng et al., 2011a,b,c, 2012).

A 16 kHz, 110 dB pure tone generated by a NI 4461 Dynamic Signal Acquisition and Generation system (National Instruments New Zealand Ltd.) was delivered to one of the ears through a closed field magnetic speaker with a tapered tip (Tucker-Davis Technologies), attached to a 3-mm cone-shaped speculum that was fitted tightly into the external auditory canal, for 1 h. This kind of stimulus has previously been reported to induce tinnitus (Tan et al., 2007; Zheng et al., 2011a, 2012). Acoustic values were calibrated before noise exposure by connecting the speaker to a 1/4-inch prepolarized free-field microphone (Type 40BE, GRAS Sound, and Vibration) via the speculum used to fit into the external auditory canal. The unexposed ear was blocked with cone-shaped foam and taped against the foam surface. The sham animals were kept under anesthesia for the same duration as the noise trauma animals, but without noise exposure.

### Auditory function

Auditory function in both ears of exposed and sham animals before and immediately after the acoustic trauma or sham treatment was measured using auditory brainstem-evoked response (ABR) thresholds in response to pure tones at 8, 16, and 20 kHz (Zheng et al., 2011a,b,c, 2012). Briefly, the animals were anesthetized as previously described and subdermal needle electrodes were placed at the vertex and over the bullae with a reference electrode at the occiput. ABR thresholds were tested for tone bursts (2 ms rise/decay, 1 ms plateau) presented at a rate of 50/s, in a decreasing intensity series, beginning with levels that elicited distinct evoked potentials. Hearing threshold was indicated by the lowest intensity that produced visually distinct potentials.

### Tinnitus assessment

The presence of tinnitus was assessed in each rat after the acoustic trauma using a conditioned lick suppression paradigm described in our previous publications (Zheng et al., 2011a,b,c, 2012). Tinnitus assessment was conducted in an operant conditioning test chamber (ENV-007, Med Associates Inc.) using a conditioned lick suppression paradigm, which we developed based on the lever pressing paradigm described by Brozoski et al. (2007). Drinking activity was measured by a lickometer with a photobeam (ENV-251L, Med Associates Inc.). A speaker (ENV-224DM, Med Associates Inc.) directly above the drinking tube produced broad band noise (BBN) or a pure tone of different frequencies and intensities via a sound generator (ANL926, Med Associates Inc.). The stimulus intensities used were 0, 30, 40, 50, 70, 80, 90, and 100 dB SPL. The BBN was white noise ranging from 3 to 20 kHz with no equalization and the level was measured using an NI 4461 Dynamic Signal Acquisition and Generation card (National Instruments New Zealand Ltd.) and was calibrated as dB (SPL). The chamber floor was lined with stainless steel rods (0.48 cm in diameter, ENV-005, Med Associates Inc.) and delivered an electric shock (0.35 mA) produced by a constant current shock source (ENV-410B, Med Associates Inc.) through a scrambler (ENV-412, Med Associates Inc.).

The conditioned lick suppression paradigm consisted of 15 min of testing every day and the animals went through three phases: the acclimation phase, the Pavlovian conditioned suppression training phase, and the frequency discrimination phase. During the acclimation phase, the BBN was played throughout the 15 min session except at 10 random intervals, at which point 15 s acoustic stimulus presentations were inserted. Two of the 10 presentations were always speaker off periods (i.e., silence) and the remaining eight were one of BBN, 10 or 20 kHz tones at one of four different intensity levels in a random order with each stimulus repeated twice within each session. The type of stimulus varied randomly between sessions, but remained constant within a session. We found that animals exposed to the acoustic trauma did not produce significantly more lick suppression than the sham animals to the 20 kHz tones as we have observed in previous studies (Zheng et al., 2011a,b,c, 2012); therefore, we tested them at higher frequencies, including 32 kHz. We found that the noise-exposed rats did show the expected downward shift in the discrimination function at 32 kHz compared with the sham rats, and therefore we substituted this higher frequency in the paradigm described above.

Following acclimation, each animal received conditioned suppression training in which a 3-s foot shock (0.35 mA) was presented at the end of each speaker off period. The foot shock acted as an unconditioned stimulus (UCS) and the speaker off period acted as a conditioned stimulus (CS). Over a few sessions, the rats reacted to the speaker off by stopping licking (i.e., the conditioned suppression). The lick suppression was measured by comparing the number of licks in the 15-s period preceding the stimulus presentation (A) and the 15-s period during the stimulus presentation (B), i.e., the SR:

$$\text{SR}=\frac{\text{B}}{\text{A + B}}$$

Once the lick suppression was established (SR < 0.2), the rats were subjected to the frequency discrimination test, during which the acoustic stimuli were presented in the same way as in the acclimation and the suppression training (i.e., BBN or pure tones of 10, 20, or 32 kHz at 0, 30, 40, 50, 70, 80, 90, or 100 dB SPL). However, the foot shock was delivered only if the SR for the speaker off period was >0.2.

If a rat did not have tinnitus, the presentation of the stimuli had no effect on its drinking activity. However, if a rat had tinnitus, the tinnitus served as the CS during the training sessions, therefore, a stimulus with sensory features resembling tinnitus during the testing session would produce greater suppression. Each stimulus was tested five to six times. Tinnitus was first assessed in these rats at 2 weeks after the noise exposure. Although it was possible that the severity of tinnitus varied over the testing period, we controlled for this by counterbalancing the order of the treatment conditions.

### Statistical analysis

All data were first tested for the normal parametric assumptions of normality and homogeneity of variance (Kutner et al., 2005). Data for the ABRs and the pre-drug condition were then analyzed using a Linear Mixed Model (LMM) analysis using a restricted maximum likelihood procedure in SPSS 19 (Kutner et al., 2005; Gurka and Edwards, 2011). LMM analyses were used as an alternative to repeated measures ANOVAs because there was extensive autocorrelation in the data; LMM analyses model the covariance structure of the repeated measures data in order to address this problem (Kutner et al., 2005; Norusis, 2010; Gurka and Edwards, 2011). The most appropriate covariance matrix structure was chosen based on the smallest Akaike’s Information Criterion (Norusis, 2010). Group (noise trauma versus sham), stimulus intensity, and the interaction between group and stimulus intensity were evaluated. While a significant group effect indicated significant differences between the noise trauma and sham groups irrespective of stimulus intensity, a significant interaction between group and intensity indicated a significant difference between groups as a function of intensity. *P* < 0.05 was considered significant.

We recognized that not all animals exposed to acoustic trauma necessarily develop tinnitus and that therefore the inclusion of animals that did not have tinnitus or had only mild tinnitus, could bias the results of the drug study (Heffner and Harrington, 2002). For this reason, we also analyzed the data for individual animals both pre-drug and following drug treatment, using the criterion that if three or more mean SR values for an individual animal were less than the mean for the sham controls, this animal could be considered to exhibit evidence of tinnitus. We then used a test for two proportions to determine whether the same animals which exhibited tinnitus in the pre-drug condition, also exhibited tinnitus in the memantine and washout conditions (Agresti, 2007; Sprent and Smeeton, 2007).

## Results

### Auditory function and the confirmation of tinnitus

**Figure 1:**
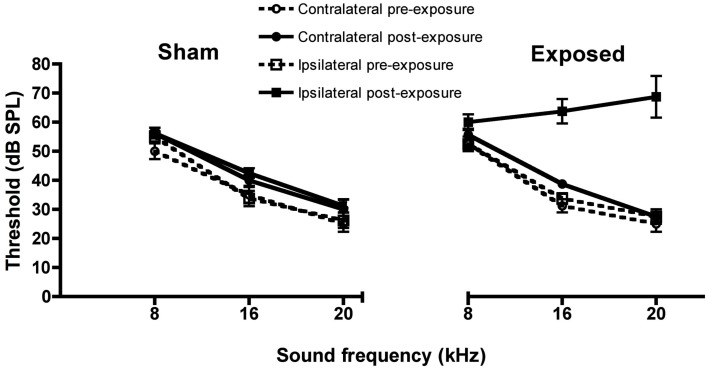
**Auditory brainstem-evoked response thresholds for the ipsilateral and contralateral ears of acoustic trauma-exposed and sham control animals before and after exposure, as a function of intensity in dB SPL and frequency in kHz**. Bars represent means ± 1 SE.

**Figure 2:**
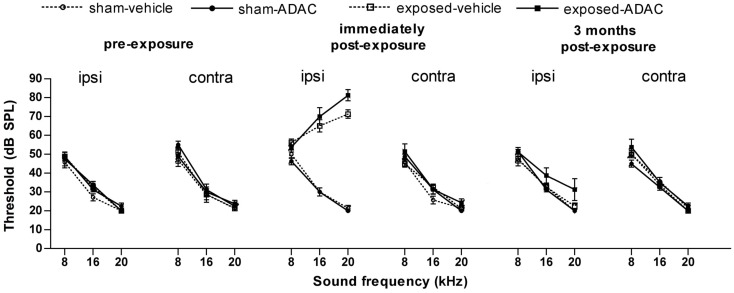
**Auditory brainstem-evoked response thresholds from another, similar experiment, in which measurements were made over 3 months following the acoustic trauma**. Data are shown for the ipsilateral and contralateral ears of acoustic trauma-exposed and sham control animals before and after exposure, as a function of intensity in dB SPL and frequency in kHz. Bars represent means ± 1 SE. The drug being tested in this study was ADAC, an adenosine amine congener (Zheng et al., unpublished observations).

Unilateral acoustic trauma produced an immediate loss of auditory function in the exposed ear at 16 and 20 kHz as indicated by the elevated ABR thresholds [*F*(1, 167) = 16.52, *P* ≤ 0.0001 for group and *F*(1, 167) = 23.74 for the group × ear × time interaction, *P* ≤ 0.0001, i.e., the affected ear in the noise-exposed group showed a significant change in the ABR threshold between the pre- and post-exposure measurements; Figure 1]. However, in this group of animals the ABR thresholds were not significantly different from sham controls at 8 kHz (Figure 1). Auditory function in the ears of sham-exposed animals and in the unexposed ears of the acoustic trauma-exposed animals, were not affected. Although we did not test ABR thresholds in these animals at the end of this experiment, in a similar experiment involving eight sham and eight acoustic trauma-exposed rats, and exactly the same methods, we found no significant differences in hearing threshold at 3 months post-exposure, despite a large and significant elevation of threshold in the ipsilateral, exposed ear immediately after the noise trauma (Figure 2; Zheng et al., unpublished observations).

**Figure 3:**
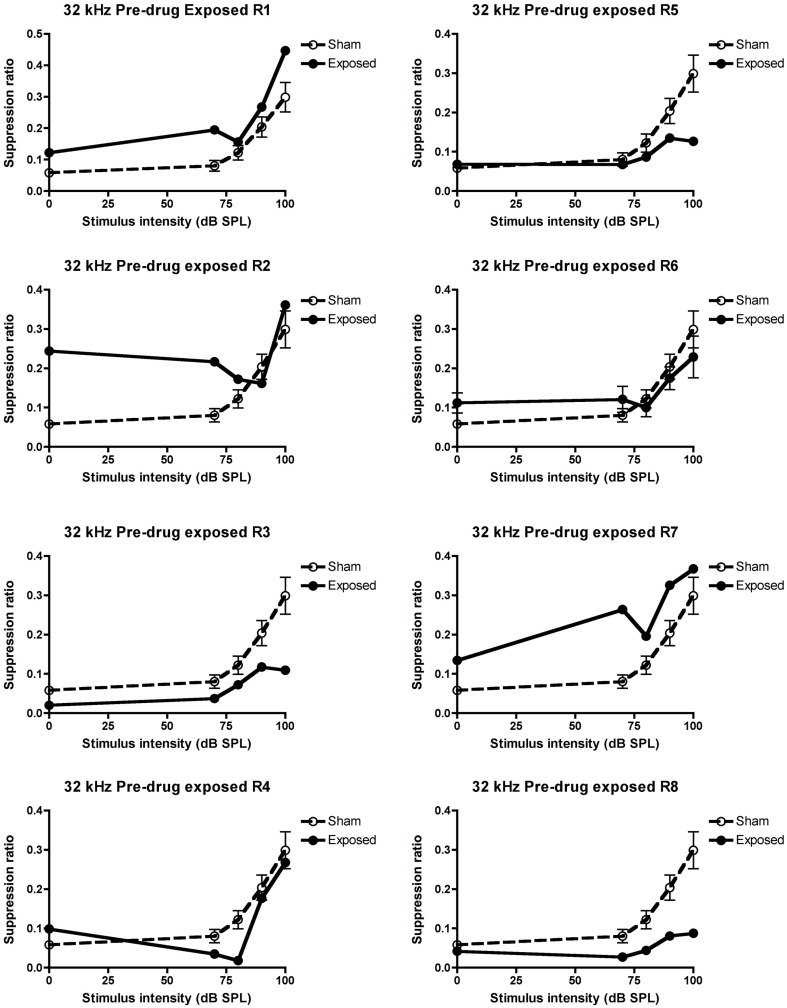
**Suppression ratios for the individual acoustic trauma-exposed animals plotted against the mean SRs of the sham control animals before any drug or vehicle administration, as a function of intensity in dB SPL to a 32 kHz stimulus**. R1–8 represent individual acoustic trauma-exposed animals. Symbols represent means ± 1 SE.

In the conditioned suppression task, as expected, stimulus intensity always had a significant effect on the SR, i.e., SR increased with the increase in stimulus intensity for all the frequencies tested, and therefore will not be discussed further. When tested under the lick suppression paradigms at 2 weeks following the acoustic trauma and before any vehicle or drug treatment, there was a significant downward shift in the SR curve for the acoustic trauma group compared to the sham controls in response to the 32 kHz tones [significant interaction between group and intensity: *F*(4, 285.09) = 2.97, *P* ≤ 0.02] but not in response to the BBN or 10 kHz tones (for either group or the interaction between group and intensity; data not shown), indicating the presence of tinnitus similar to a 32-kHz tone. When the data for the individual animals was inspected, five out of eight of them met the criterion of at least three mean SR values that were lower than the sham control values, and therefore these animals (R3, 4, 5, 6, and 8) were considered to have tinnitus. Their individual data are shown in Figure 3.

After the confirmation of tinnitus in the pre-drug testing, the animals were injected with the saline vehicle every day while being tested for tinnitus. The results were similar following administration of the saline vehicle: the SR was significantly smaller in the acoustic trauma group in response to the 32-kHz tones at higher stimulus intensities [interaction between group and intensity: *F*(4, 344.351) = 3.44, *P* ≤ 0.009; data not shown] but not the 10 kHz tones (for either group or the interaction between group and intensity). Although there was no significant group effect for the BBN, there was a significant interaction between treatment and intensity [*F*(4, 334.85) = 3.8, *P* ≤ 0.005]; however, this was due to the higher SRs for the noise-exposed animals at low stimulus intensities (data not shown).

### Effects of memantine

**Figure 4:**
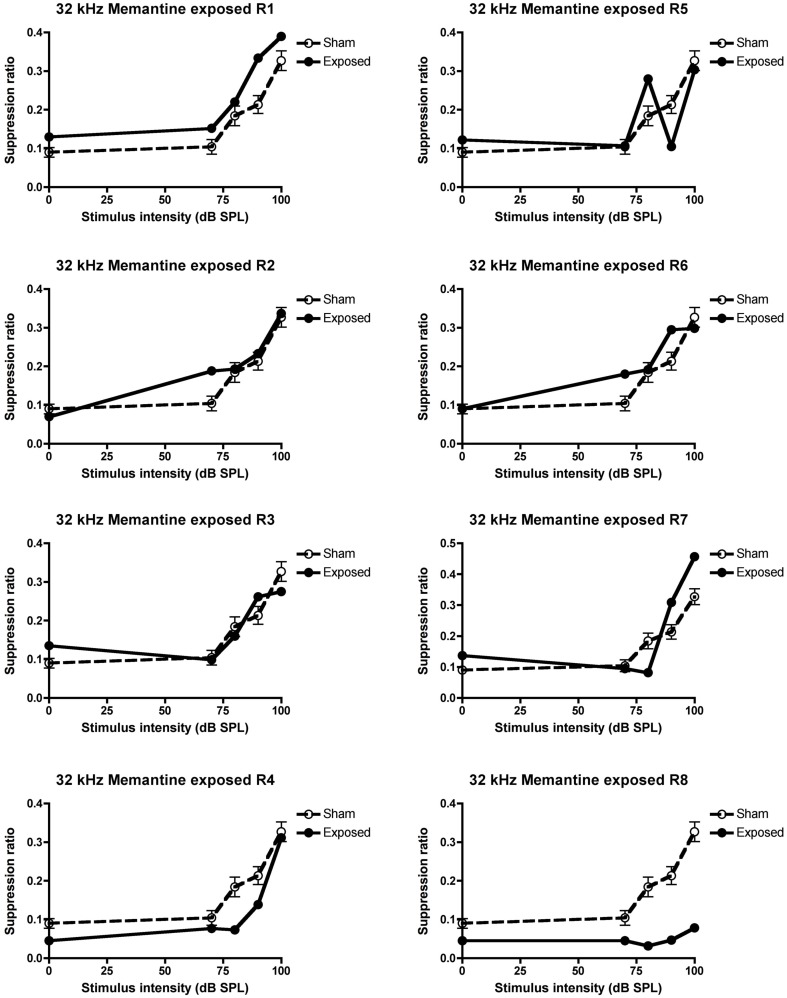
**Suppression ratios for the individual acoustic trauma-exposed animals plotted against the mean SRs of the sham control animals following the 5 mg/kg s.c. memantine administration, as a function of intensity in dB SPL to a 32 kHz stimulus**. R1–8 represent individual acoustic trauma-exposed animals. Symbols represent means ± 1 SE.

When treated with 5 mg/kg memantine, only two of the five animals that exhibited tinnitus in the pre-drug condition, continued to exhibit it, i.e., only two of the same five animals (R4 and 8) that had at least three SR means lower than the sham control condition, continued to fulfill that criterion. This was a significant decrease in the proportion of animals with tinnitus, which had initially exhibited evidence of tinnitus (*z* = 2.74, *P* ≤ 0.006; see Figure 4 for the data for the individual animals).

**Figure 5:**
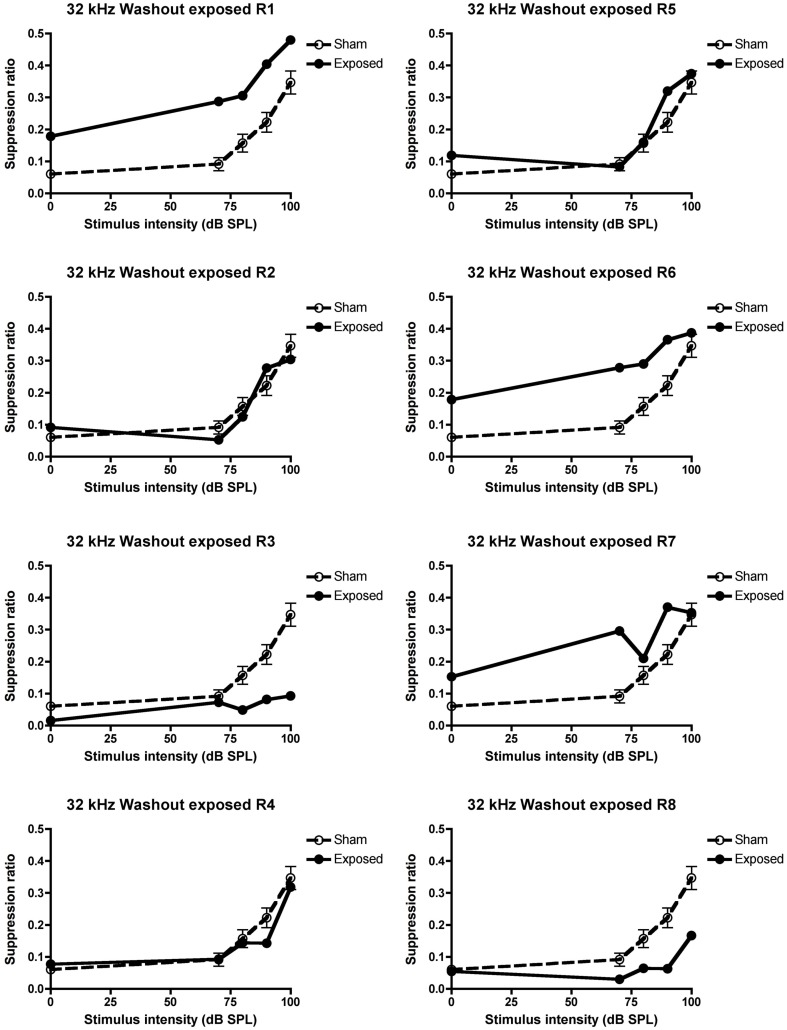
**Suppression ratios for the individual acoustic trauma-exposed animals plotted against the mean SRs of the sham control animals following the washout of the 5 mg/kg s.c. memantine administration, as a function of intensity in dB SPL to a 32 kHz stimulus**. R1–8 represent individual acoustic trauma-exposed animals. Symbols represent means ± 1 SE.

In order to ensure that the loss of behavioral signs of tinnitus in these animals was not due to the tinnitus merely disappearing by itself over time, the animals were given a washout period during which no drug treatment took place. During this period, three of the five animals which exhibited tinnitus in the pre-drug condition, continued to exhibit it (R3, 4, and 8) and this proportion was not significantly different from the pre-drug condition (see Figure 5 for the individual data).

## Discussion

Consistent with our previous studies (Zheng et al., 2011a, 2012), we found that an acoustic trauma stimulus consisting of a 16 kHz pure tone, at 110 dB SPL, delivered unilaterally for 1 h, produced a large and significant increase in the ABR threshold in the affected ear. However, in contrast to our previous studies, this shift occurred only for tones at 16 and 20 kHz and not for 8 kHz. We also observed a significant frequency specific decrease in the average SR in a conditioned lick suppression paradigm, in response to 32 kHz tones but not in response to 10 kHz tones or BBN. Five out of eight animals tested fulfilled the criterion of at least three mean SR values in response to 32 kHz tones being less than in the sham control condition and therefore these five animals were considered to exhibit evidence of tinnitus. In contrast to our previous studies (Zheng et al., 2011a, 2012), we found no change in the SR discrimination curve at 20 kHz. Memantine, at a dose of 5 mg/kg s.c, significantly reduced the number of animals exhibiting tinnitus from five to two, which then increased to 3/5 during the washout period.

It is unclear to us why the same acoustic trauma produced less severe hearing loss at a lower frequency range (i.e., 8 kHz) when compared with our previous studies (Zheng et al., 2011a, 2012). It could be due to slight variations in the position of the speculum inserted in the external auditory canal. Nonetheless, this degree of damage was enough to produce the behavioral signs of tinnitus in 5/8 exposed animals. It is interesting to note that the frequency specific downward shift in the SR curve was at 32 kHz rather than the 20 kHz observed previously (Zheng et al., 2011a, 2012). Following noise trauma-induced hearing loss, the auditory cortex undergoes reorganization and neurons in the hearing loss frequency region begin to respond to frequencies of the neighboring neurons (Roberts et al., 2010). Therefore, with no ABR threshold changes at 8 kHz in the present study, it is possible that the hearing loss was more pronounced at higher frequencies (i.e., >20 kHz as tested), which resulted in a perception of tinnitus at frequencies higher than 20 kHz. Nonetheless, we observed a clear reduction in the SRs in 5/8 acoustic trauma-exposed animals responding specifically to 32 kHz tones.

It is difficult, if not impossible, to completely exclude the possibility that some aspect of the change in SR that we used to quantify tinnitus-related behavior, might be due to hearing loss caused by the acoustic trauma. We did not test long-term ABR thresholds in these animals at the end of this experiment. However, in a similar experiment involving eight sham and eight acoustic trauma-exposed rats, employing exactly the same acoustic trauma stimulus, we found no significant differences in hearing threshold at 3 months post-exposure, despite large and significant elevations of hearing threshold in the ipsilateral, exposed ear immediately after the noise trauma (Figure 2; Zheng et al., unpublished observations). This result suggests that, using our acoustic trauma stimulus, ABR thresholds gradually return toward normal over time. Since these animals exhibited behavioral evidence of confirmed tinnitus for about 2 months before the memantine treatment, this makes it less likely that the SR changes that we observed during the memantine treatment period were due simply to hearing loss.

Following the 5 mg/kg memantine treatment 1 h before each tinnitus testing session, we observed a significant attenuation of the shift in the SR curve at 32 kHz in 3/5 animals that exhibited tinnitus in the pre-drug condition. This suggests that the memantine significantly reduced tinnitus in the acoustic trauma-exposed animals that developed tinnitus. This is the first evidence that systemic administration of memantine after the confirmation of tinnitus can effectively reduce tinnitus in an animal model. Lobarinas et al. (2006) reported that it had no significant effect on salicylate- and quinine-induced tinnitus in rats, or on the increase in cortical auditory-evoked potentials caused by salicylate (see also Lobarinas et al., 2008). However, drug- and noise-induced tinnitus are not necessarily comparable in terms of their mechanisms or response to drug treatments (see Eggermont and Roberts, 2004; Eggermont, 2005; Kaltenbach, 2006; Roberts et al., 2010 for reviews). Furthermore, Lobarinas et al. used lower doses (1.5 and 3 mg/kg/day) of memantine than we used in the current study (5 mg/kg). We did not conduct a dose-response analysis in this case because of the expense of memantine. Using the dose adjustment calculation employed by the FDA to calculate human equivalent doses (Reagan-Shaw et al., 2007), our dose of 5 mg/kg is approximately equivalent to 56.83 mg/day for a 70 kg human adult. In the only published clinical trial, Figueiredo et al. (2008) found no significant effect of 20 mg memantine per day on patients’ tinnitus compared to placebo. It is conceivable that higher doses could have had a greater effect on the patients’ tinnitus. However, adverse side effects may also have developed with higher doses.

Despite the previous evidence that memantine has little effect on tinnitus, Guitton and Dudai (2007) reported that acoustic trauma-induced tinnitus could be prevented by cochlear application of the NMDA receptor polyamine site antagonist, ifenprodil. However, this study showed that ifenprodil was only effective when administered within the first 4 days of noise trauma but not after tinnitus was “consolidated.” In the present study, we showed that memantine was effective in reversing confirmed tinnitus in 3/5 animals at least 4 weeks after the acoustic trauma. The main difference between our study and Guitton and Dudai’s is the drug administration route. It is well accepted that although tinnitus can be initiated by noise trauma-induced hair cell damage, once it is established it is generated by hyperactive neurons in the brain (Roberts et al., 2010). It is possible that at an early stage, blocking NMDA receptors in the cochlea is enough to prevent the further development of tinnitus; once long-term tinnitus has developed, blockade of NMDA receptors in the central nervous system is necessary. Some beneficial effect on tinnitus patients has also been reported with neramexane, which is also an NMDA receptor antagonist with additional actions at the α_9_α_10_ nicotinic acetylcholine and 5-HT_3_ receptors (Suckfull et al., 2011). This clinical trial, along with the current evidence, suggests that memantine and similar drugs may be worthy of further consideration for the treatment of tinnitus.

In order to test the reversibility of memantine’s effects on tinnitus, we evaluated the animals again during a washout period. Following the washout period, the SRs for the 32 kHz stimulus were again lower than the sham control condition in 3/5 animals. This proportion was not statistically different from that of the pre-drug condition and therefore provided some evidence that the tinnitus had returned in these animals following the washout of memantine. However, the result would have been more convincing if all five animals had again exhibited evidence of tinnitus, as they did in the pre-drug condition. The fact that this did not happen could be interpreted as a long-lasting effect of the drug on tinnitus, or could have been due to the washout period not being sufficiently long for the drug to be entirely eliminated, or merely because the tinnitus had disappeared over time by itself. It is very unlikely that the drug had not been entirely eliminated from the system given that the washout period lasted for 15 days; the half-life of a single dose of memantine in humans is about 65 h and the pharmacokinetics are very similar following multiple-doses for 14 days (Liu et al., 2008). It is also unlikely that tinnitus had disappeared by itself, because tinnitus had been confirmed in these animals in two separate testing periods, the pre-drug and the vehicle conditions. In other words, these animals had confirmed tinnitus for about 2 months before the memantine treatment. In our previous experiments, the longest time point when the exposed animals had confirmed tinnitus was at 10 months after the acoustic trauma (Zheng et al., 2011a), therefore, it seems unlikely that tinnitus would disappear in these animals after 2 months. However, we cannot exclude the possibility that the severity of the tinnitus fluctuated over time and the present study was not designed to investigate the longitude of memantine’s effect on tinnitus. Further studies are required to explore this issue.

It has been reported that NMDA receptor antagonists, such as memantine, can impair associative learning (Schugens et al., 1997; Zajaczkowski et al., 1997). Therefore, it could be argued that the lack of difference in the frequency discrimination curve between the sham and exposed animals following memantine treatment was due to impaired associative learning produced by memantine. However, if this was the case, the animals would not have been able to produce lick suppression in response to all of the stimulus intensities tested and the frequency discrimination curves would be similar and shifted close to 0.5 across the stimulus intensity range. Moreover, the sham animals also received the same dose of memantine as the exposed animals. If memantine impaired the conditioned lick suppression response, there should have been a significant change in the frequency discrimination curve before and after the drug treatment in the sham animals. However, this was not the case.

In conclusion, this study provides the first evidence that memantine may reduce tinnitus in rats caused by acoustic trauma. It may therefore be worth further investigating the potential therapeutic effects of memantine and similar drugs in the treatment of human tinnitus.

## Conflict of Interest Statement

The authors declare that the research was conducted in the absence of any commercial or financial relationships that could be construed as a potential conflict of interest.

## Abbreviations

SR, suppression ratio; ABR, auditory brainstem-evoked response; SPL, sound pressure level; BBN, broad band noise.

## References

[B1] Agresti A. (2007). Introduction to Categorical Data Analysis. New Jersey: Wiley

[B2] Bauer C. A. Brozoski T. J. (2001). Assessing tinnitus and prospective tinnitus therapeutics using a psychophysical animal model. J. Assoc. Res. Otolaryngol. 2, 54–64 11545150 10.1007/s101620010030 PMC3201094

[B3] Brozoski T. J. Spires T. J. D. Bauer C. A. (2007). Vigabatrin, a GABA transaminase inhibitor, reversibly eliminates tinnitus in an animal model. J. Assoc. Res. Otolarnygol. 8, 105–118 10.1007/s10162-006-0067-2 PMC2538419 17221143

[B4] Darlington C. L. Smith P. F. (2007). Drug treatments for tinnitus. Prog. Brain Res. 166, 249–262 10.1016/S0079-6123(07)66023-3 17956789

[B5] Dong S. Mulders W. H. Rodger J. Woo S. Robertson D. (2010). Acoustic trauma evokes hyperactivity and changes in gene expression in guinea-pig auditory brainstem. Eur. J. Neurosci. 31, 1616–1628 20525074 10.1111/j.1460-9568.2010.07183.x

[B6] Eggermont J. J. (2005). Tinnitus: neurobiological substrates. Drug Discov. Today 10, 1283–1290 10.1016/S1359-6446(05)03542-7 16214672

[B7] Eggermont J. J. Roberts L. E. (2004). The neuroscience of tinnitus. Trends Neurosci. 27, 676–682 10.1016/j.tins.2004.08.010 15474168

[B8] Elgoyhen A. B. Langguth B. (2011). “Pharmacological approaches to tinnitus treatment,” in Textbook of Tinnitus, eds Moller A. R. Langguth B. DeRidder D. Kleinjung T. (Heidelberg: Springer), 625–637

[B9] Figueiredo R. R. Langguth B. Mello de Oliveira P. Aparecida de Azvedo A. (2008). Tinnitus treatment with memantine. Otolaryngol. Head Neck Surg. 138, 492–496 10.1016/j.otohns.2007.11.027 18359360

[B10] Guitton M. J. Caston J. Ruel J. Johnson R. M. Pujol R. Puel J. L. (2003). Salicylate induces tinnitus through activation of cochlear NMDA receptors. J. Neurosci. 23, 3944–3952 12736364 10.1523/JNEUROSCI.23-09-03944.2003 PMC6742173

[B11] Guitton M. J. Dudai Y. (2007). Blockade of cochlear NMDA receptors prevents long-term tinnitus during a brief consolidation window after acoustic trauma. Neural Plast. 80904 10.1155/2007/80904 18301716 PMC2246076

[B12] Gurka M. J. Edwards L. J. (2011). “Mixed models,” in Essential Statistical Methods for Medical Statistics, eds Rao C. R. Miller J. P. Rao D. C. (Amsterdam: Elsevier), 146–173

[B13] Heffner H. E. Harrington I. A. (2002). Tinnitus in hamsters following exposure to intense sound. Hear. Res. 170, 83–95 10.1016/S0378-5955(02)00343-X 12208543

[B14] Kaltenbach J. A. (2006). The dorsal cochlear nucleus as a participant in the auditory, attentional and emotional components of tinnitus. Hear. Res. 216, 224–234 10.1016/j.heares.2006.01.002 16469461

[B15] Kutner M. H. Nachtsheim C. J. Neter J. Li W. (2005). Applied Linear Statistical Models. Boston: McGraw-Hill Irwin

[B16] Leaver A. M. Renier L. Chevillet M. A. Morgan S. Kim H. J. Rauschecker J. P. (2011). Dysregulation of limbic and auditory networks in tinnitus. Neuron 69, 33–43 10.1016/j.neuron.2010.12.002 21220097 PMC3092532

[B17] Lin X. (1997). Action potentials and underlying voltage-dependent currents studied in cultured spiral ganglion neurons of the postnatal gerbil. Hear. Res. 108, 157–179 10.1016/S0378-5955(97)00050-6 9213129

[B18] Liu M. Y. Meng S. N. Wu H. Z. Wang S. Wei M. J. (2008). Pharmacokinetics of single-dose and multiple-dose memantine in healthy Chinese volunteers using an analytic method of liquid chromatography-tandem mass spectrometry. Clin. Ther. 30, 641–653 10.1016/j.clinthera.2008.04.005 18498913

[B19] Lobarinas E. Sun W. Stolzberg D. Jianzhong L. Salvi R. (2008). Human brain imaging of tinnitus and animal models. Semin. Hear. 24, 333–349 10.1055/s-0028-1095893 19122834 PMC2613289

[B20] Lobarinas E. Yang G. Sun W. Ding D. Mirza N. Dalby-Brown W. Hilczmayer E. Fitzgerald S. Zhang L. Salvi R. (2006). Salicylate- and quinine-induced tinnitus and effects of memantine. Acta Otolaryngol. Suppl. 126, 13–19 10.1080/03655230600895408 17114137

[B21] Middleton J. W. Kiritani T. Pedersen C. Turner J. G. Shepherd G. M. Tzounopoulos T. (2011). Mice with behavioral evidence of tinnitus exhibit dorsal cochlear nucleus hyperactivity because of decreased GABAergic inhibition. Proc. Natl. Acad. Sci. U.S.A. 108, 7601–7606 10.1073/pnas.1100223108 21502491 PMC3088638

[B22] Moller A. R. (2000). Similarities between severe tinnitus and chronic pain. J. Am. Acad. Audiol. 11, 115–124 10755808

[B23] Mulders W. H. Robertson D. (2011). Progressive centralization of midbrain hyperactivity after acoustic trauma. Neuroscience 192, 753–760 10.1016/j.neuroscience.2011.06.046 21723924

[B24] Norusis M. J. (2010). PASW18 Statistics 18 Advanced Statistical Procedures Companion. New Jersey: Prentice Hall

[B25] Olivares D. Deshpande V. K. Shi Y. Lahiri D. K. Greig N. H. Rogers J. T. Huang X. (2012). *N*-Methyl d-Aspartate (NMDA) receptor antagonists and memantine treatment for Alzheimer’s disease, vascular dementia, and Parkinson’s disease. Curr. Alzheimer Res. 9, 746–758 21875407 10.2174/156720512801322564 PMC5002349

[B26] Rauschecker J. P. Leaver A. M. Mühlau M. (2010). Tuning out the noise: limbic-auditory interactions in tinnitus. Neuron 66, 819–826 10.1016/j.neuron.2010.04.032 20620868 PMC2904345

[B27] Reagan-Shaw S. Nihal M. Ahmad N. (2007). Dose translation from animal to human studies revisited. FASEB J. 22, 659–661 10.1096/fj.07-9574LSF 17942826

[B28] Roberts L. E. Eggermont J. J. Caspary D. M. Shore S. E. Melcher J. R. Kaltenbach J. A. (2010). Ringing ears: the neuroscience of tinnitus. J. Neurosci. 30, 14972–14979 10.1523/JNEUROSCI.4879-10.2010 21068300 PMC3073522

[B29] Schugens M. M. Egerter R. Daum I. Schepelmann K. Klockgether T. Löschmann P. A. (1997). The NMDA antagonist memantine impairs classical eyeblink conditioning in humans. Neurosci. Lett. 224, 57–60 10.1016/S0304-3940(97)13452-8 9132691

[B30] Shargorodsky J. Curhan G. C. Farwell W. R. (2010). Prevalence and characteristics of tinnitus among US adults. Am. J. Med. 123, 711–718 10.1016/j.amjmed.2010.02.015 20670725

[B31] Sprent P. Smeeton N. C. (2007). Applied Nonparametric Statistical Methods. Boca Raton: Chapman and Hall/CRC

[B32] Suckfull M. Althaus M. Ellers-Lenz B. Gebauer A. Gortelmeyer R. Jastreboff P. J. Moebius H. J. Rosenberg T. Russ H. Wirth Y. Krueger H. (2011). A randomized, double-blind, placebo-controlled clinical trial to evaluate the efficacy and safety of neramexane in patients with moderate to severe subjective tinnitus. BMC Ear Nose Throat Disord. 11, 1 10.1186/1472-6815-11-1 21223542 PMC3031239

[B33] Tan J. Rüttiger L. Panford-Walsh R. Singer W. Schulze H. Kilian S. B. Hadjab S. Zimmermann U. Köpschall I. Rohbock K. Knipper M. (2007). Tinnitus behavior and hearing function correlate with the reciprocal expression patterns of BDNF and Arg3.1/arc in auditory neurons following acoustic trauma. Neuroscience 145, 715–726 10.1016/j.neuroscience.2006.11.067 17275194

[B34] Vogler D. P. Robertson D. Mulders W. H. (2011). Hyperactivity in the ventral cochlear nucleus after cochlear trauma. J. Neurosci. 31, 6639–6645 10.1523/JNEUROSCI.6538-10.2011 21543592 PMC6632868

[B35] Wang H. Brozoski T. J. Caspary D. M. (2011). Inhibitory neurotransmission in animal models of tinnitus: maladaptive plasticity. Hear Res. 9, 111–117 10.1016/j.heares.2011.04.004 21527325 PMC3172385

[B36] Zajaczkowski W. Frankiewicz T. Parsons C. G. Danysz W. (1997). Uncompetitive NMDA receptor antagonists attenuate NMDA-induced impairment of passive avoidance learning and LTP. Neuropharmacology 36, 961–971 10.1016/S0028-3908(97)00070-1 9257940

[B37] Zheng Y. Hamilton E. Stiles L. McNamara E. de Waele C. Smith P.F. Darlington C. L. (2011a). Acoustic trauma that can cause chronic tinnitus impairs impulsive control but not performance accuracy in the 5-choice serial reaction time task in rats. Neuroscience 180, 75–84 10.1016/j.neuroscience.2011.02.040 21352899

[B38] Zheng Y. Hamilton E. Begum S. Smith P. F. Darlington C. L. (2011b). The effects of acoustic trauma that can cause tinnitus on spatial performance in rats. Neuroscience 186, 48–56 10.1016/j.neuroscience.2011.04.052 21549180

[B39] Zheng Y. Hamilton E. McNamara E. Smith P. F. Darlington C. L. (2011c). The effects of chronic tinnitus caused by acoustic trauma on social behaviour and anxiety in rats. Neuroscience 193, 143–153 10.1016/j.neuroscience.2011.07.026 21782007

[B40] Zheng Y. Vagal S. McNamara E. Darlington C. L. Smith P. F. (2012). A dose-response analysis of the effects of l-baclofen on chronic tinnitus caused by acoustic trauma in rats. Neuropharmacology 62, 940–946 10.1016/j.neuropharm.2011.09.027 22005094

